# Treatment outcomes and prognostic factors in nonmetastatic metaplastic breast cancer patients: a multicenter retrospective cohort study

**DOI:** 10.2340/1651-226X.2024.40413

**Published:** 2024-08-04

**Authors:** Mirosława Püsküllüoğlu, Aleksandra Konieczna, Katarzyna Świderska, Joanna Streb, Małgorzata Pieniążek, Aleksandra Grela-Wojewoda, Renata Pacholczak-Madej, Anna Mucha-Małecka, Jerzy W. Mituś, Joanna Szpor, Michał Kunkiel, Agnieszka Rudzińska, Michał Jarząb, Marek Ziobro

**Affiliations:** a Department of Clinical Oncology, Maria Sklodowska-Curie National Research Institute of Oncology, Krakow, Poland; b Department of Breast Cancer and Reconstructive Surgery, Maria Sklodowska-Curie National Research Institute of Oncology, Warsaw, Poland; c Breast Cancer Unit, Maria Skłodowska-Curie National Research Institute of Oncology, Gliwice, Poland; d Department of Oncology, Jagiellonian University Medical College, Krakow, Poland; e Department of Oncology, Wrocław Medical University, Wrocław, Poland; f Lower Silesian Comprehensive Cancer Center, Wrocław, Poland; g Department of Anatomy, Jagiellonian University Medical College, Krakow, Poland; h Department of Gynaecological Oncology, Maria Sklodowska-Curie National Research Institute of Oncology, Krakow Branch, Krakow, Poland; i Department of Chemotherapy, The District Hospital, Sucha Beskidzka, Poland; j Department of Radiotherapy, Maria Sklodowska-Curie National Research Institute of Oncology, Krakow, Poland; k Department of Surgical Oncology, Maria Sklodowska-Curie National Research Institute of Oncology, Krakow, Poland; l Department of Pathomorphology, Jagiellonian University Medical College, Kraków, Poland; m Department of Oncology. Grochowski Hospital, Warsaw, Poland

**Keywords:** Metaplastic breast cancer, systemic treatment, chemotherapy, disease-free survival, overall survival

## Abstract

**Background and purpose:**

Metaplastic breast carcinoma (BC-Mp) is an uncommon subtype that poses unique challenges. The limited information on patient prognosis and therapeutic strategies motivated our research initiative. We aimed to assess disease-free survival (DFS), overall survival (OS), and influential factors in patients with nonmetastatic BC-Mp.

**Materials and methods:**

In this multicenter retrospective cohort study, clinicopathological data for nonmetastatic BC-Mp patients treated at four oncology units in Poland (2012–2022) were gathered.

**Results:**

Among 115 women (median age 61, range: 28–91), the median tumor size was 40 mm (range 20–130); 30% of patients exhibited positive local lymph nodes. The majority of patients presented with stage II (46%) and triple-negative breast cancer (TNBC) (84%). Radiotherapy was administered to 61% of patients. Surgical procedures included breast-conserving surgery in 31% of patients and mastectomy in 68%. Eighty-three per cent of patients received chemotherapy. The median estimated DFS and OS were 59 and 68 months, respectively. Multivariable analysis revealed that tumor size influenced DFS and OS (Hazard ratios [HR] = 1.02, 95%CI 0.01–0.03 for both endpoints) and taxanes application improved DFS (HR = 0.47, 95%CI 0.24–0.93), but other factors did not. For patients receiving neoadjuvant systemic therapy (*N* = 51), taxanes improved DFS and OS according to univariable analysis.

**Interpretation:**

Our findings highlight poor DFS and OS regardless of receiving optimal treatment, emphasizing the need for tailored therapeutic strategies for BC-Mp patients. Taxanes appear promising in a neoadjuvant setting, particularly within the current standard of care for the TNBC subtype.

## Introduction

The guidelines for the treatment of early breast cancer (BC) are well established and primarily depend on molecular BC subtypes [1]. The molecular phenotype of the tumor, substituted by the immunohistochemical phenotype, serves as a valuable guide in therapeutic decision-making. However, it is most reliably proven among the most common morphological subtypes, such as invasive ductal carcinoma (IDC), representing 80% of histopathological diagnoses for invasive BC and invasive lobular carcinoma, representing approximately 10% of them [2]. Less frequent subtypes, such as metaplastic carcinoma (BC-Mp), occur in less than 1% of all invasive BCs.

BC-Mp is characterized by various combinations of adenocarcinoma with mesenchymal and epithelial components. Immunohistochemical staining revealed increased expression of markers of epithelial‐mesenchymal transition and cancer stem cells [3]. All these distinct histopathological characteristics create a hetereogenous group, which is classified by the World Health Organization (WHO) into low‐grade adenosquamous carcinoma, fibromatosis‐like metaplastic carcinoma, squamous cell carcinoma, spindle cell carcinoma, and carcinoma with mesenchymal differentiation (chondroid, osseous, and other types of mesenchymal differentiation) [4]. BC-Mp is mostly diagnosed as a triple negative tumor (90% of cases) [5]. The variety of BC-Mp pathomorphologies translates into clinical aspects. Compared to IDC, MpBC is usually diagnosed at a more advanced stage and has a worse prognosis than IDC with a similar stage and grade [5]. According to some studies [6–8], BC-Mp is reported to exhibit diminished chemosensitivity. Ongoing debates persist, especially concerning prognostic factors and treatment guidelines, attributable to the diverse and rare characteristics of BC-Mp. A significant number of patients with initially localized disease ultimately encounter either metastatic spread or local recurrence. Limited data exist on treatment outcomes, especially in the neoadjuvant setting. Available retrospective studies present significant limitations commonly lacking information about regimens employed [9–12], including patients treated more than 20 years ago [12–14] or gathering small populations [13–16], while prospective trials are missing [16]. Gathering extensive real-world data from diverse healthcare systems is crucial for robust evidence, especially for indications lacking clinical trial support. It captures diverse populations, varied settings, and long-term outcomes, supporting guideline development and improving patient care.

The objective of this investigation was to assess disease-free survival (DFS), overall survival (OS), and contributing factors in individuals diagnosed with nonmetastatic BC-Mp who underwent treatment at four cancer reference centra/university hospitals in Poland.

## Materials and methods

### Patients and study design

We organized a retrospective cohort investigation in accordance with the Strengthening the Reporting of Observational Studies in Epidemiology (STROBE) (Supplementary Materials, Supplementary Table S1) guidelines involving nonmetastatic BC-Mp patients who underwent treatment between 2012 and 2022 at four reference oncology departments: the Maria Sklodowska-Curie National Research Institute of Oncology Branch in Warsaw, Krakow, and Gliwice, as well as the Department of Oncology at the University Hospital in Krakow, Poland.

Patients diagnosed with BC-Mp were identified through the hospitals’ registry systems. The inclusion criterion for the study was individuals with a confirmed diagnosis of BC-Mp indicated in either postsurgical or core biopsy pathology reports. The standard approach for confirming the diagnosis of BC-Mp involves a combination of morphological assessment and immunohistochemical staining [17]. The absence of an authentic pathology report, a diagnosis of cancer spread, or concurrent active malignancies served as exclusion criteria for participation in the study. The study did not impose any restrictions based on the patients’ sex or age.

The tumor was considered ER and PR positive if at least 1% of invasive tumor cells showed nuclear staining [18]. HER-2 expression was assessed via immunohistochemistry (IHC) and scored from 0 to 3: 0 for no or weak-moderate incomplete staining in ≤ 10% of cells, 1 for weak incomplete staining in > 10%, 2 for weak-moderate in > 10% or strong in < 10%, and 3 for strong complete staining in 10% of cells. Cases with a HER-2 score of 2 underwent further fluorescence *in situ* hybridization (FISH) analysis [19].

### Data collection

The data collected included age, sex, comorbidities, menopausal status, history of other malignancies, tumor stage (including tumor size and local lymph node involvement), primary tumor location, dates and types of systemic treatment, radiotherapy and surgery, survival status, dates of local recurrence and/or cancer dissemination, dates of the last visit, and histopathological information (comprising histology, ER, PR, HER-2, Ki-67 status, presence of ductal carcinoma *in situ* [DCIS], tumor grade, lymphovascular invasion [LV], and the presence of different BC-Mp components).

### Statistical analysis

The analyses were performed using R software, version 4.3.2, with the significance level set at 0.05. Descriptive statistics, including the means, standard deviations (SDs), medians, quartiles, and ranges, are presented for quantitative variables. For qualitative variables, absolute and relative frequencies (*N* and %) were documented.

Univariable and multiple logistic regression were employed to model the potential impact of predictors on a dichotomous variable. ORs (odds ratios) and 95% confidence intervals (CIs) are presented.

Univariable and multiple Cox regression (proportional hazards model) were employed to model the potential impact of predictors on a time to event. Hazard ratios (HRs), alongside the 95% CIs, are presented.

The selection of independent variables was determined by their significance in univariable analyses, with consideration given to ensuring that the subjects per variable (SPV) or events per variable (EPV) exceeded 10 or at least 5 in cases where reaching 10 was unattainable. Multicollinearity was assessed using the variance inflation factor (VIF), and predictors exhibiting VIF values exceeding 5 were systematically excluded from the model.

### Ethical considerations

Approval for this study’s ethical considerations was provided by the Ethical Committee at the Maria Sklodowska-Curie National Research Institute of Oncology Branch Krakow, as denoted by decision number 3/2023 dated 18 April 2023.

## Results

### Population characteristics

The study comprised a cohort of 115 female patients. The median age at diagnosis of BC-Mp was 61 years (interquartile range: 48–71), with a mean age of 60 years (SD: 15.7), spanning an age range of 28–91 years. BC-Mp represented less than 1% of the overall BC cases in each hospital registry.

The median size of the tumors measured 40 mm (interquartile range: 25–60), with a mean of 46.2 mm (SD: 29.1), ranging from 20 to 130 mm (data unavailable for 1 patient). *N* = 35 patients (30%) exhibited a positive status for local lymph nodes. The median Ki67 expression level was 50% (interquartile range: 35–70), and the mean was 51% (SD: 25.3), varying from 3% to 100% (data unavailable for 6 patients). Additional clinicopathological details can be found in Table 1.

**Table 1:** Patients’ clinical and pathological data.

Parameter		Total (*N* = 115) (%)
**Side**	Left	67 (58.3)
	Right	48 (41.7)
**HR status**	Negative	101 (87.8)
	Positive	14 (12.2)
**HER2 status**	Negative	111 (96.5)
	Positive	4 (3.5)
**Subtype**	TNBC	97 (84.4)
	Luminal B	14 (12.2)
	HER-2 positive	4 (3.5)
**Presurgery histopathology carried in a reference cancer centra**	No	23 (20.0)
	Yes	92 (80.0)
**Grade**	G2	24 (20.9)
	G3	85 (73.9)
	Unknown	6 (5.2)
**LV**	Negative	47 (40.9)
	Positive	9 (7.8)
	Unknown	59 (51.3)
**DCIS component**	Absent	81 (70.4)
	Present	33 (28.7)
	Unknown	1 (0.9)
Type of component*	NST	39 (33.9)
	Squamous	46 (40.0)
	Spindle cell/pleomorphic/sarcomatid	30 (26.1)
	Osseous/chondroid	23 (20.0)
	Mesenchymal unspecified	6 (5.2)
	Lipid rich	1 (0.9)
**Initial diagnosis**	Self-diagnosis	76 (66.1)
	Diagnostic imaging	33 (28.7)
	Unknown	6 (5.2)
**Menopause**	No	32 (27.8)
	Yes	83 (72.2)
**TNM stage**	Stage 1	22 (19.1)
	Stage 2	53 (46.1)
	Stage 3	39 (33.9)
	Stage 4	0 (0.0)
	Unknown	1 (0.9)
**Secondary neoplasm in patient’s history**	No	78 (67.8)
	Yes	17 (14.8)
	Unknown	20 (17.4)

ICH: immunohistochemistry; DCIS: ductal carcinoma *in situ*; FISH: fluorescence *in situ* hybridization; HER2: human epidermal growth factor receptor 2; HR: hormonal receptor; LV: lymphovascular invasion; NST: no special type; TNM: tumor, node, metastasis (8th edition).

* Any mesenchymal or epithelial component detailed in the histopathology report.

### Treatment received

The treatment modalities (surgery, radiotherapy, and systemic therapies) received by the patients are presented in Table 2. Tables 3 and 4 present more detailed characteristics of the neoadjuvant and adjuvant systemic treatment cohorts.

**Table 2:** Details of treatment received by the patients.

Parameter		Total (*N* = 115) (%)
**Radiotherapy**	No	43 (37.4)
	Yes	70 (60.9)
	Unknown	2 (1.7)
**Surgical procedure**	Breast conserving surgery	36 (31.3)
	Mastectomy	78 (67.8)
	Unknown	1 (0.9)
**Chemotherapy**	No	20 (17.4)
	Yes	95 (82.6)
**All planned treatment received**	No	38 (33.0)
	Yes	68 (59.1)
	Unknown	9 (7.8)
**Chemotherapy setting**	Neoadjuvant	51 (44.4)
	Adjuvant	37 (32.2)
	Sandwich*	4 (3.4)
	Unknown	3 (2.6)
	Not applicable	20 (17.4)
Type of systemic therapy received**,***	Anthracycline based	73 (63.5)
	Taxane based	72 (62.6)
	Platinum based	30 (26.1)
	Capecitabine	16 (13.9)
	Hormonal agents****	12 (10.4)

* Adjuvant capecitabine assessed into ‘neoadjuvant group’.

** Could be more than one agent.

*** 58 (50.4%) patients received anthracyclines and taxanes in different combinations.

**** All patients received also chemotherapy.

**Table 3:** Characteristics of the neoadjuvant systemic treatment cohort.

Parameter		Total (*N* = 51) (%)
**Subtype**	TNBC	49 (96.1)
	Luminal B	2 (3.9)
	HER2* positive	0 (0)
**Presurgery histopathology carried in a reference cancer centra**	No	12 (23.5)
	Yes	39 (76.5)
**Grade**	G2	12 (23.5)
	G3	35 (66.7)
	Unknown	5 (9.8)
**TNM stage**	Stage 1	2 (3.9)
	Stage 2	24 (47.1)
	Stage 3	25 (49.0)
**Radiotherapy**	No	17 (33.3)
	Yes	33 (64.7)
	Unknown	1 (0.8)
**Surgical procedure**	Breast-conserving surgery	14 (27.5)
	Mastectomy	36 (67.8)
	Unknown	1 (2.0)
**Full planned treatment received**	No	19 (37.3)
	Yes	31 (60.8)
	Unknown	1 (2.0)
**Type of systemic therapy received**	Anthracycline based	41 (80.4)
	Taxane based	39 (62.6)
	Taxane and athracycline based	33 (64.7)
	Platinum based	21 (41.2)
	Capecitabine*	16 (31.4)
	Hormonal agents**	2 (3.9)
**Pathological complete response**	No, without progression	36 (70.6)
	No, with progression	3 (5.9)
	Yes	10 (19.6)
	No data	2 (3.9)

* Postoperation.

** All patients received also chemotherapy.

ICH: immunohistochemistry; DCIS: ductal carcinoma *in situ*; FISH: fluorescence *in situ* hybridization; HER2: human epidermal growth factor receptor 2; HR: hormonal receptor; LV: lymphovascular invasion; NST: no special type; TNM: tumor, node, metastasis (8th edition).

**Table 4:** Characteristics of the adjuvant systemic treatment cohort.

Parameter		Total (*N* = 37) (%)
**Subtype**	TNBC	33 (89.2)
	Luminal B	4 (10.8)
	HER-2 positive	0 (0)
**Presurgery histopathology carried in a reference cancer centra**	No	8 (21.6)
	Yes	29 (78.4)
**Grade**	G2	8 (21.6)
	G3	28 (75.7)
	Unknown	1 (2.7)
**TNM stage**	Stage 1	16 (43.2)
	Stage 2	15 (40.5)
	Stage 3	5 (13.5)
	Unknown	1 (2.7)
**Radiotherapy**	No	11 (29.7)
	Yes	25 (67.6)
	Unknown	1 (2.7)
**Surgical procedure**	Breast-conserving surgery	20 (54.1)
	Mastectomy	17 (46.0)
	Unknown	0 (0.0)
**Full planned treatment received**	No	4 (10.8)
	Yes	32 (86.5)
	Unknown	1 (2.7)
**Type of systemic therapy received**	Anthracycline based	28 (75.7)
	Taxane based	27 (73.0)
	Taxane AND anthracycline based	21 (16.2)
	Platinum based	6 (26.1)
	Hormonal agents*	5 (13.5)

ICH: immunohistochemistry; FISH: fluorescence *in situ* hybridization; HER2: human epidermal growth factor receptor 2; HR: hormonal receptor; TNM: tumor, node, metastasis (8th edition).

* All patients received also chemotherapy

The correlation between tumor size and local lymph node involvement was statistically significant for the whole population. Each additional millimeter in tumor size increased the odds of lymph node involvement by 1.6% (OR = 1.016; 95% CI = 1.002–1.03; *p* = 0.023).

### Disease-free survival and overall survival

**Figure 1:**
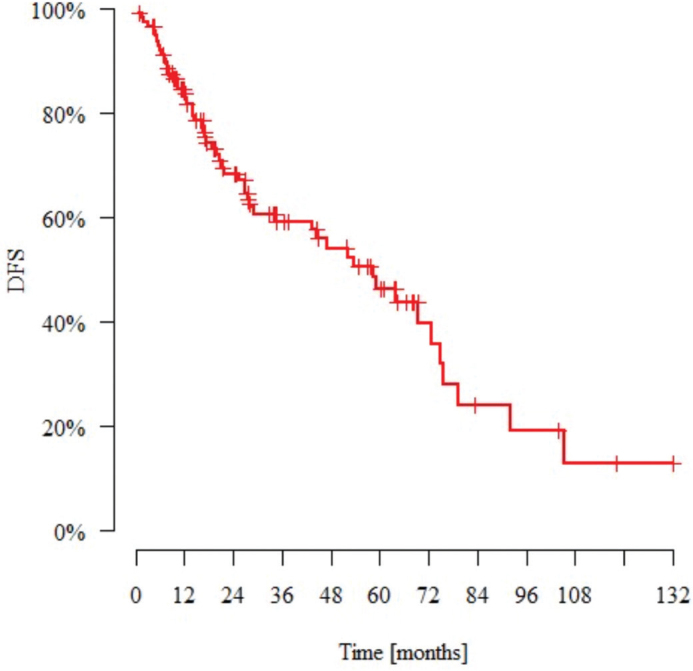
Kaplan–Meier estimate of disease-free survival for patients with nonmetastatic metaplastic breast cancer. DFS: disease-free survival.

**Figure 2:**
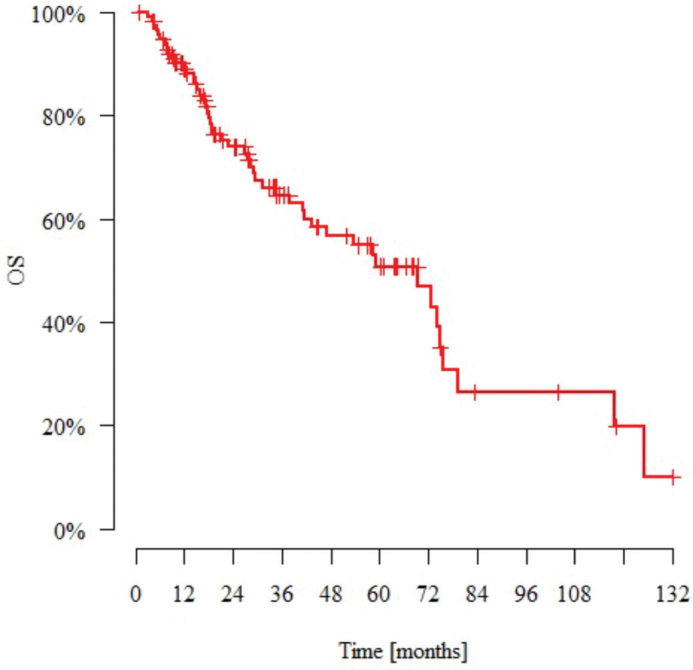
Kaplan–Meier estimate of overall survival for patients with nonmetastatic metaplastic breast cancer. OS: overall survival.

The median observation time was 27.4 months (range: 0.8–132.0 months). *N* = 49 patients (43%) died and *N* = 22 (19%) had metastasis during the follow-up period. The median time to metastasis (from the day of diagnosis) was 15.2 months. *N* = 12 patients (10%) experienced local recurrence during this time. The median time to recurrence (from the day of diagnosis) was 14.6 months. Tables 5 and 6 present the DFS and OS data. Figures 1 and 2 present Kaplan–Meier estimate of DFS and OS, respectively.

**Table 5:** Estimated disease-free survival data for metaplastic breast cancer patients.

Patients	Events	Disease-free survival			
		12 months	36 months	60 months	Median [months]
115	54	84.6%	59.1%	46.3%	58.6

**Table 6:** Estimated overall survival data for metaplastic breast cancer patients.

Patients	Events	Overall survival			
		12 months	36 months	60 months	Median [months]
115	49	89.1%	64.7%	50.8%	69.4

### Factors influencing disease-free survival

According to the univariable proportional hazards Cox model for the whole population, (1) each successive millimeter increase in tumor size increases the probability of distant metastases, local recurrence, or death at any given time by 1.8% (HR = 1.018, 95%CI 0.009–0.027); (2) every additional year of life increases the probability of distant metastases, local recurrence, or death at any given time by 1.9% (HR = 1.019, 95%CI 0.001–0.038); (3) performing histopathological examination at one of the cancer reference centra reduces the probability of distant metastases, local recurrence, or death at any given time by 46.3% (HR = 0.537, 95%CI 0.294–0.980); (4) LV positivity increases the probability of distant metastases, local recurrence, or death at any given time by 3.13 times (HR = 3.131, 95%CI 1.192–8.226); (5) receiving systemic treatment decreases the probability of distant metastases, local recurrence, or death at any given time by 67.0% (HR = 0.33, 95%CI = 0.174–0.626); and (6) the use of taxanes decreases the probability of distant metastases, local recurrence, or death at any given time by 53.7% (HR = 0.463, 95%CI = 0.270–0.795). The multivariable proportional hazards Cox model showed that (1) each successive millimeter increase in tumor size increased the probability of distant metastasis, local recurrence, or death at any given time by 1.9% (HR = 1.017, 95%CI 1.01–1.028); (2) performing histopathological examination at one of the cancer reference centra reduced the probability of distant metastasis, local recurrence, or death at any given time by 58.9% (HR = 0.411, 95%CI 0.215–0.784); and (3) taxane usage reduced the probability of distant metastasis, local recurrence, or death at any given time by 53% (HR = 0.47, 95%CI 0.237–0.932). These findings are summarized in Table 7. The presence of different BC-Mp histological components did not influence DFS.

**Table 7:** Results of univariable and multivariable Cox proportional hazards models for disease-free survival.

Variable		*N*	Events	Univariable models				Multivariable model			
				HR	95%CI		*p*	HR	95%CI		*p*
Local lymph nodes involved	No	80	38	1	ref.						
	Yes	35	16	0.755	0.419	1.361	0.35				
Tumor size [mm]		-	-	1.018	1.009	1.027	<0.001 *	1.019	1.01	1.028	<0.001 *
Age [years]		-	-	1.019	1.001	1.038	0.043 *	1.012	0.992	1.034	0.234
Primary tumor side	Left	67	32	1	ref.						
	Right	48	22	1.201	0.693	2.079	0.514				
HR status	Negative	101	49	1	ref.						
	Positive	14	5	0.75	0.298	1.889	0.541				
HER2 status	Negative	111	52	1	ref.						
	Positive	4	2	2.913	0.68	12.487	0.15				
Subtype	TNBC	97	47	1	ref.						
	Luminal	14	5	0.773	0.306	1.952	0.586				
	HER2 positive	4	2	2.821	0.656	12.137	0.163				
Ki67 [%]		-	-	0.993	0.981	1.005	0.263				
Initial histopathology carried in a reference centra	No	23	15	1	ref.			1	ref.		
	Yes	92	39	0.537	0.294	0.98	0.043 *	0.411	0.215	0.784	0.007 *
Grade	G2	24	8	1	ref.						
	G3	85	43	1.279	0.597	2.74	0.526				
LV	Negative	47	23	1	ref.						
	Positive	9	6	3.131	1.192	8.226	0.021 *				
Diagnosis	Self-diagnosis	76	34	1	ref.						
	Diagnostic imaging	33	16	0.97	0.531	1.77	0.92				
Menopause	No	32	15	1	ref.						
	Yes	83	39	1.061	0.584	1.926	0.846				
TNM stage	Stage 1	22	6	1	ref.						
	Stage 2	53	26	2.433	0.997	5.934	0.051				
	Stage 3	39	21	2.447	0.986	6.073	0.054				
	Stage 4	0	0	1	ref.						
Systemic treatment	No	20	13	1	ref.			1	ref.		
	Yes	95	41	0.33	0.174	0.626	0.001 *	0.69	0.29	1.64	0.401
Radiotherapy	No	43	17	1	ref.						
	Yes	70	36	0.95	0.531	1.699	0.863				
Full planned therapy received	No	38	21	1	ref.			1	ref.		
	Yes	68	27	0.279	0.15	0.519	<0.001 *	0.364	0.172	0.774	0.009 *
Anthracyclines	No	42	19	1	ref.						
	Yes	73	35	0.737	0.42	1.296	0.29				
Taxanes	No	43	27	1	ref.			1	ref.		
	Yes	72	27	0.463	0.27	0.795	0.005 *	0.47	0.237	0.932	0.031*
Anthracyclines + Taxanes	No	57	29	1	ref.						
	Yes	58	25	0.694	0.403	1.193	0.186				
Platins	No	85	47	1	ref.						
	Yes	30	7	0.498	0.224	1.106	0.087				
Capecitabine	No	99	52	1	ref.						
	Yes	16	2	0.354	0.085	1.469	0.153				
Systemic therapy setting	Neoadjuvant	51	23	1	ref.						
	Adjuvant	37	19	0.614	0.321	1.174	0.14				
Surgical procedure	Breast-conserving surgery	36	18	1	ref.						
	Mastectomy	78	36	1.206	0.682	2.135	0.519				
Secondary neoplasm	No	78	45	1	ref.						
	Yes	17	8	0.993	0.465	2.118	0.985				

CI: confidence interval; ER: estrogen receptor, HER2: human epidermal growth factor receptor 2; HR: hazard ratio; N: number; TNBC: triple-negative breast cancer.

* statistically significant (*p* < 0.05).

For patients receiving neoadjuvant chemotherapy (NAC) (*N* = 51 patients), the univariable proportional hazards Cox model showed that the use of taxanes reduced the likelihood of distant metastases, local recurrence, or death at any given time by 68.6% (HR = 0.314, 95%CI 0.115–0.854). For patients receiving adjuvant treatment (*N* = 37 patients), these data were not confirmed.

### Factors influencing overall survival

Considering the entire population in a univariable proportional hazards Cox model analysis, it was noted that (1) each additional millimeter in primary tumor size increased the probability of death at any given time by 2.1% (HR = 1.021, 95%CI 1.011–1.030); (2) each additional year of age increased the probability of death at any given time by 2.3% (HR = 1.023, 95%CI 1.004–1.043); (3) the LV-positive score increased the probability of death at any given time by 4.023 times (HR = 4.023, 95%CI 1.491–10.855); (4) chemotherapy decreased the probability of death at any given time by 69.2% (HR = 0.308, 95%CI 0.158–0.601); and (5) the use of taxanes decreased the probability of death at any given time by 51.2% (HR = 0.488, 95%CI 0.278–0.859). The occurrence of local recurrence did not influence OS (*p* = 0.247). The multivariable proportional hazards Cox model indicated that each additional millimeter increase in primary tumor size increased the likelihood of death at any given time by 2.1% (HR = 1.021, 95%CI 1.012–1.031). The data are summarized in Table 8. The presence of different BC-Mp histological components did not influence OS.

**Table 8:** Results of univariable and multivariable proportional hazards Cox models for overall survival.

Variable		*N*	Deaths	Univariable models				Multiple model			
				HR	95%CI		*p*	HR	95%CI		*p*
Lymph nodes involved	No	80	33	1	ref.						
	Yes	35	16	0.924	0.507	1.684	0.796				
Tumor size [mm]		-	-	1.021	1.011	1.03	<0.001 *	1.021	1.012	1.031	<0.001*
Age [years]		-	-	1.023	1.004	1.043	0.018 *	1.018	0.996	1.039	0.108
Primary tumor side	Left	67	28	1	ref.						
	Right	48	21	1.235	0.697	2.189	0.47				
HR status	Negative	101	46	1	ref.						
	Positive	14	3	0.438	0.136	1.413	0.167				
HER2 status	Negative	111	47	1	ref.						
	Positive	4	2	2.629	0.624	11.072	0.188				
Subtype	TNBC	97	44	1	ref.						
	Luminal	14	3	0.45	0.139	1.454	0.182				
	HER-2 positive	4	2	2.429	0.576	10.249	0.227				
Ki67 [%]		-	-	0.992	0.979	1.004	0.2				
Initial histopathology carried in a reference centra	No	23	14	1	ref.						
	Yes	92	35	0.54	0.289	1.01	0.054				
Grade	G2	24	8	1	ref.						
	G3	85	38	1.081	0.501	2.334	0.843				
LV	Negative	47	22	1	ref.						
	Positive	9	6	4.023	1.491	10.855	0.006 *				
Diagnosis	Self-diagnosis	76	33	1	ref.						
	Diagnostic imaging	33	13	0.854	0.446	1.638	0.635				
Menopause	No	32	13	1	ref.						
	Yes	83	36	1.207	0.638	2.283	0.563				
TNM stage	Stage 1	22	6	1	ref.						
	Stage 2	53	21	1.821	0.73	4.545	0.199				
	Stage 3	39	21	2.473	0.996	6.14	0.051				
	Stage 4	0	0	1	ref.						
Chemotherapy	No	20	12	1	ref.			1	ref.		
	Yes	95	37	0.308	0.158	0.601	0.001 *	0.663	0.264	1.517	0.305
Radiotherapy	No	43	16	1	ref.						
	Yes	70	32	0.886	0.483	1.625	0.695				
Full planned therapy received	No	38	21	1	ref.			1	ref.		
	Yes	68	23	0.214	0.111	0.41	<0.001 *	0.253	0.114	0.561	0.001 *
Anthracyclines	No	42	17	1	ref.						
	Yes	73	32	0.795	0.44	1.439	0.449				
Taxanes	No	43	25	1	ref.			1	ref.		
	Yes	72	24	0.488	0.278	0.859	0.013 *	0.62	0.307	1.253	0.183
Anthracyclines + Taxanes	No	57	27	1	ref.						
	Yes	58	22	0.692	0.391	1.226	0.207				
Platins	No	85	42	1	ref.						
	Yes	30	7	0.587	0.262	1.316	0.196				
Capecitabine	No	99	47	1	ref.						
	Yes	16	2	0.456	0.109	1.901	0.281				
Systemic therapy setting	Neoadjuvant	51	21	1	ref.						
	Adjuvant	37	17	0.585	0.297	1.154	0.122				
Surgical procedure	Breast-conserving surgery	36	16	1	ref.						
	Mastectomy	78	33	1.242	0.679	2.27	0.481				
Secondary neoplasm	No	78	41	1	ref.						
	Yes	17	7	1.062	0.473	2.385	0.883				

CI: confidence interval; ER: estrogen receptor; HER2: human epidermal growth factor receptor 2; HR: hazard ratio; N: number; TNBC: triple-negative breast cancer.

* statistically significant (p < 0.05)

For patients receiving NAC (*N* = 51 patients), the univariable Cox proportional hazards model again showed that the application of taxanes reduced the probability of death at any given time by 67.9% (HR = 0.321, 95%CI 0.118–0.873). The use of different types of chemotherapy in the adjuvant setting (*N* = 37) did not affect OS (*p* < 0.05).

### Factors influencing pathological complete response after neoadjuvant treatment

For individuals who underwent NAC (*N* = 51 patients), the univariable Cox proportional hazards model indicated that a pathological complete response (pCR) was not related to any of the studied factors: tumor size, presence of pathological local lymph nodes, stage (I-III), HR positivity, menopausal status, Ki67 level, histological components, type of chemotherapy, or receiving fully planned systemic treatment (all *p* > 0.05).

### Factors influencing the diagnosis of secondary malignancy

None of the patients’ characteristic parameters correlated with a diagnosis of secondary malignancy in their medical history, as all *p* values were greater than 0.05 (patients with active malignancies at the time of BC with metastatic progression diagnosis were excluded from the study).

## Discussion

In this study, we presented real-world treatment data for 115 patients with nonmetastatic BC-Mp. The proportion of BC-Mp among the entire BC population was less than 1% according to our demographic data, which generally corresponds with data from other populations [5, 10, 12]. Studies regarding patients with BC-Mp, who received systemic treatment have been presented in Table 9. In the available literature, the majority of BC-Mp patients presented with TNBC [5, 6, 9, 10, 12, 21–23], a large primary tumor size [5, 7, 10, 15], no lymph node involvement [5, 7, 10, 12, 21], poor differentiation [12, 15, 21–23], and stage 2 disease [9, 23, 24], which aligns with the results from our study. Few studies have reported on Ki67, but the available ones show a high Ki67 status [23]. There are also limited data regarding LV; however, in our study, the frequency of positive status was markedly lower than that in other studies [25]. Reports are also ambiguous in terms of prevalent histology, with some claiming that squamous [15, 23] or other mesenchymal [13] components or mixed subtypes [25] are the most frequent. In the present study, with each additional millimeter in tumor size, there was an increased likelihood of lymph node involvement. However, in our previous study involving a population of 45 nonmetastatic BC-Mp patients who underwent initial surgical treatment, such a correlation was not demonstrated [26]. It is plausible that this correlation, which has also been confirmed by other studies [27], could only be evident in larger tumors or with a larger patient cohort.

**Table 9:** Literature regarding systemic (neo)adjuvant treatment in nonmetastatic metaplastic breast cancer patients.

Ref.	*N* (years treated) Study type	IHC/BC subtype	Pathology	Staging	Systemic neo(adjuvant) treatment	Outcomes	Conclusions
[25]	217 (2010–2017) Retrospective cohort study	HR neg 74.7% HER neg 93.5% HR-/HER2- (69.6) HR +/HER2- (24.0%) HR-/HER2+ (5.1%) HR +/HER2+ (1.4%)	Spindle *n* = 27 Squamous *n* = 33 Carcinoma with mesenchymal differentiation *n* = 59 Mixed squamous *n* = 53 Mixed spindle *n* = 45	0 (0.5%) I (22.1%) II (63.1%) III `(14.3%) T0 (0.5%), T1 (29%), T2 (62.2%), T3 (6.9%), T4 (1.4%) N0 (71.9%), N1 (17.1%), N2 (5.1%), N3 (6%)	CTH yes *n* = 203/217 (NAC *n* = 16) Tx+A 69 (31.8%) A−Tx 85 (39.2%) Tx/A 25 (11.5%) Platinum-containing 12 (5.5%) Others 12 (5.5%) ET yes *n* = 29 AniHER2 yes *n* = 8	pCR *n* = 1/16 (6.3%) SD/PD = 9/16 (56.3%) -> changed treatment to platinum-containing regimen as a 2^nd^ line. Adj CTH: Tx+A had prolonged RFS and BCSS than Tx/A (*p* = 0.003 and *p* = 0.014), Other regimens (including X, MAID and unknown regimens) had shorter RFS and BCSS compared to Tx+A (*p* = 0.004 and *p* = 0.002). A−Tx or platinum-containing regimens had inferior RFS compared to the Tx+A, but without statistical significance (*p* = 0.093 and *p* = 0.052). No survival differences in BCSS between A–Tx or platinum-containing regimens and Tx+A (*p* = 0.348 and *p* = 0.297)	Independent prognostic factors in the multivariate COX analysis: - AJCC stage, - mixed subtype, - chemotherapy. Mixed BC-Mp: - exhibit a higher level of aggressiveness compared to pure forms, - are associated with shorter survival outcomes compared to their pure counterparts.
[10]	1,605 (2010–2018) Retrospective cohort study	ER neg 77.8% PrR neg 86.4% HER2 neg 93.3%, HR+/HER2-*n* = 401 HR+/HER2+ *n* = 37 HR-/HER2+ *n* = 71 HR-/HER2-*n* = 1,096	Lack of data about exact pathology according to WHO classification	I (22.4%); II (57.4%); III `(15.3%) T1 (24.9%), T2 (47.9%), T3 (16.9%), T4 (10.3%) N0 (76.2%), N1 (17.2%), N2 (4.4%), N3 (2.2%)	CTH yes *n* = 1,084, CTH no *n* = 521 Lack of data about chemotherapy regimens	mFU was 53 mo (1–107 mo). 3-year and 5-year OS rates were 74.5 and 67.4%.	In multivariate Cox regression analysis, - patients’ age - T, N, and M stage - surgery and radiation therapy were all independent prognostic factors for OS. Chemotherapy was not an independent prognostic factor for BC-Mp patients. BC-Mp is more aggressive than IDC.
[9]	205 (2010–2021) Case series	ER neg 89.4% PrR neg 90% HER2 neg 91.1% No subtype classification	Spindle *n* = 2 Mixed *n* = 21 Squamous *n* = 10 Matrix producing *n* = 4	I (9.8%) II (58.5%) III (25.4%) No exact data about T and N stage	CTH yes *n* = 152 CTH no *n* = 24 Lack of data about chemotherapy regimens	The mOS was 66 (12–118) mo, and the mDFS was 56.8 (11–102) mo. Multivariate Cox regression analysis revealed that surgical treatment was associated with decreased risk of death (HR 0.11, 95% CI 0.02–0.54, *p* = 0.01) while advanced TNM stage was associated with increased risk of death (HR 1.5, 95% CI 1.04–2.28, *p* = 0.03).	The surgical treatment and TNM stage were the only independent risk factors related to patients’ OS.
[12]	155 (1994–2019) Retrospective cohort study	ER neg 87.1% PrR neg 94.19% HER2 neg 91.61% No subtype classification	Lack of data about exact pathology according to WHO classification	T1 (27.10%), T2 (65.81%), T3 (6.45%), T4 (0.65%) N0 (80%), N1 (16.13%), N2 (3.23%), N3 (0.65%) Lack of data about AJCC stage	No NAC CTH yes *n* = 140 CTH no *n* = 11 Lack of data about chemotherapy regimens ET yes *n* = 21 ET no *n* = 131	The median FU 68 mo (range 3–277) DFS 78.06% HR 2.893% (95% CI 1.929–4.340) OS 85.16% HR 3.839% (95%CI 2.289–6.439)	Multivariable Cox regression analysis indicated that BC-Mp was an independent prognostic factor for DFS (HR = 2.240; 95% CI, 1.476–3.399, *p* = 0.0002) and OS (HR = 1.969; 95% CI, 1.147–3.382, *p* = 0.0140). Survival analysis revealed no significant difference between BC-Mp and IDC patients in DFS (HR = 1.465; 95% CI, 0.882–2.432, *p* = 0.1398) or OS (HR = 1.542; 95% CI, 0.875–2.718, *p* = 0.1340) after propensity-score matching.
[21]	81 (2005–2018) Retrospective cohort study	ER neg 80.2% PrR neg 84% HER2 neg 71.6%, TNBC *n* = 55, HR+/HER2-*n* = 20, HR+/-/HER2+ *n* = 6	Spindle *n* = 2 Carcinoma with mesenchymal differentiation *n* = 15 Low-grade adeno-squamous *n* = 2 Metaplastic carcinoma, NOS *n* = 19 Admixed with ADC *n* = 27 Squamous *n* = 16	T1-2 (64.2%), T3 (35.8%), N0 (64.2%), N+ (34.6%), Nx (1.2%) Lack of data about AJCC stage	CTH yes *n* = 75/81 (NAC *n* = 33) NAC administered according to the NSABP-B27 protocol (A- and Tx-based; 4 cycles each, every 21 days). The predominant adj CTH regimen: FEC, followed by DXL Other regimens: AC with taxane; AC; FAC. ET yes *n* = 19, no *n* = 4 83.3% of patients HER-2 positive (*n* = 5/6) received TZB	After NAC: PD *n* = 14 SD *n* = 6 PR *n* = 12 pCR *n* = 2 Five-year OS was 66.0%. On multivariate analysis: adjuvant radiotherapy correlated with better OS (*p* < 0.0001), and tumor size >5 cm, nodal involvement and LVI correlated with worse OS, (*p* = 0.019, *p* = 0.021, *p* = 0.028, respectively).	The independent predictors of improved OS: - adjuvant radiotherapy, - tumor size less or equal 5 cm, - node-negative disease, LVI absence.
[16]	39 (2015–2022) Prospective interventional study	only TNBC No exact data about IHC	Matrix producing *n* = 18 Spindle *n* = 14 Squamous *n* = 5 Mixed spindle/matrix producing *n* = 2	I (12.8%) II (71.8%) III `(15.4%) T1 (18%), T2 (56.4%), T3 (18%), T4 (7.7%) N0 (84.6%), N+ (15.4%)	All MpBC pts received NAC based on AC, if response 2 part of NAC PXL-based, if no response there was offered available clinical trial (*n* = 10)	After NAC: pCR *n* = 9 PD/SD during AC *n* = 7 progression to metastatic disease *n* = 1 A trend toward higher pCR rates of patients with MpBC of squamous histology compared to those with spindle, matrix-producing or mixed, although the statistical significance was not achieved.	In case of BC-Mp sequential A- and Tx-based NAC should be considered.
[11]	1,137 (2010–2016) Retrospective cohort study	ER neg 78.89% PrR neg 86.46% HER2 neg 94.28% Luminal A *n* = 286 Luminal B *n* = 23 HER2positive *n* = 42 TNBC *n* = 786	Lack of data about exact pathology according to WHO classification	I (23.83%) II (60.77%) III (15.39%) T/01 (26.56%), T2 (50.04%), T3 (16.71%), T4 (6.68%) N0 (77.22%), N1 (16.18%), N2 (4.75%), N3 (1.84%)	Adj only CTH yes *n* = 775; no *n* = 362 Lack of data about CTH regimens	The 5-year cumulative incidence of BCSD showed similar outcomes in both the CTH and No-CTH groups (21.1 vs. 24.3%, *p* = 0.57). Chemotherapy showed no apparent association with BCSD (HR, 1.07; 95% CI, 0.72–1.60; *p* = 0.72), even after subgroup analysis or PSM. The identified significant factors for dissemination: - race, - tumor size, - lymph node status, and radiation.	The use of CTH did not improve BCSS in the operable BC-Mp. Authors suggest that a decreased necessity for current chemotherapy should be accepted to prevent overtreatment of patients with BC-Mp.
[14]	18 (1991–2014) Retrospective case series	TNBC *n* = 13, HER2-positive *n* = 1, lack of other information	Spindle *n* = 6 Squamous *n* = 4 Chondroid *n* = 2 Sarcomatoid *n* = 1 Matrix producing *n* = 5	T1 (0%), T2 (39%), T3 (50%), T4 (11%), N0 (61%, N1 (17%), N2 (11%), N3 (11%) Data about AJCC stage are not available.	AC (*n* = 1), AC/Tx (*n* = 3), AC/Tx/P (*n* = 8), Tx/P-based regimens (*n* = 4), Tx/CTX (*n* = 1) and Tx/TZB (*n* = 1).	5/18 (28%) progressed on initial treatment including two who developed metastatic disease during NAC. The overall pCR rate was 2/18 (11%) on ACdd+Txdd and TxP+ACdd therapy.	BC-Mp is poorly responsive to NAC, with a pCR rate (11%), that is lower than expected in a predominantly TNBC cohort. BC-Mp patients with resectable disease should undergo definitive operative management.
[27]	97 (2007–2017) Retrospective cohort study	ER neg 80% PrR neg 89% HER2 neg 92% Lack of data about subtype	Spindle *n* = 18 Squamous *n* = 21 Matrix producing *n* = 31 Admixed with NST *n* = 24 Other *n* = 3	T1 (26%), T2 (44%), T3/4 (28%), unknown (2%), N0 (64%), N1 (22%), N2 (3%) Lack of data about AJCC stage.	CTH yes *n* = 63 (NAC *n* = 29); no *n* = 31 ET yes *n* = 9 The most commonly administered NAC regimen was FAC used in 16/29 (55.1%) patients.	The mFU of 39 months, 26 patients (27%) recurred (24 distant and 2 loco-regional). The OS rate of the cohort was 66% (64/97). 5 (17%) showed a pCR. Of these 5 cases, 3 were pure matrix producing, 1 mixed matrix-producing and no special type, and 1 pure spindle cell carcinoma. All tumors resulting in pCR were ER/PrR negative, one was HER2+ and received targeted neoadj regimen, and all five showed a high Ki-67 labeling index (50% or higher).	Administration of radiation therapy and chemotherapy were both associated with improved OS (HR of 0.300 for radiation, *p* value: 0.041, 95% CI: 0.095–0.950; HR of 0.397 for CTH, *p* value: 0.039, 95% CI: 0.165–0.954) without a significant effect on RFS.
[13]	46 (1992–2013) Retrospective cohort study	ER neg 91% PrR neg 87% HER2 neg 93.5% Lack of data about subtype.	Mesenchymal differentiation = 17 squamous *n* = 12 spindle *n* = 14 mixed type *n* = 3	I (4.7%) II (11.8%) III (90.3%) T1 (4.2%), T2 (11.1%), T3 (26.3%), T4(40%), N0 (8.6%), N+ (7.2%)	CTH yes *n* = 41 (neoCTH *n* = 10) CTH no *n* = 4 ET yes *n* = 6; no *n* = 35 CTH regimens: 36/46 (78.6%) AC with or without PXL/DXL, 2/46 CMF, 1/46 a sarcoma regimen -MAID; 3/46 carboplatin based CTH, 2/46 G, 1/46 TZB.	The mFU for MpBC patients was 41 mo (range 3 to168). Disease recurrence occurred in 14 (30%) patients with MpBC. Death due to disease occurred in 12 of 42 (28.6%).	The variables that had significant effect on both DFS and OS were - T-stage, - lymph node status, - neoadj therapy and RT. CTH had only a borderline effect on the DFS but not the OS.
[22]	113 (2002–2013) Retrospective cohort study	ER neg 80% PrR neg 90% HER2 neg 88% TNBC 67%	Spindle *n* = 3, Squamous *n* = 51 Matrix producing *n* = 22, Other histology (e.g. sarcoma, not otherwise specified) *n* = 27	T1 (27%), T2 (60%), T3 (8%), T4 (4%), Tx (3%) N0 (83%), N1 (13%), N2 (2%), Nx (2%) No exact AJCC stage	CTH yes *n* = 85 (NAC *n* = 23) CTH no *n* = 28 various chemotherapy regimens	pCR = 9/23 (39%).	- Adjuvant radiation correlated with LRR in multivariate analysis. - No significant correlations with LRR were found for chemotherapy use, surgery type (BCT/mastectomy), LRR, T/N stage. - CTH, DM, or LRR when based on the type of surgical resection a patient received were not significant for OS.
[7]	90 (2000–2014) Retrospective cohort study	Lack of data about IHC, luminal A *n* = 2, luminal B *n* = 17 HER2-overexpression *n* = 7 TNBC *n* = 64 Basal-like *n* = 58	Spindle *n* = 31 Squamous *n* = 28 mesenchymal differentiation *n* = 22 Mixed *n* = 5 Fibromatosis like *n* = 4	I (13.3%) II (75.6%) III `(11.1%) T1 (21.1%), T2 (56.7%), T3 (21.1%), T4 (1.1%) N0 (79.2%), N+ (20.8), no lymph node information was available in 13 of cases	CTH yes *n* = 74 (NAC *n* = 3) CTH no *n* = 16 Adj CTH regimen: PXL + A (*n* = 74) NAC: PXL+Epi or Epi+CTX+DXL *n* = 4 (no reduction of tumor size) ET *n* = 9 Adj TZB *n* = 1	The mFU was 59 mo (range 1–173) A 67.9% 5-year DFS and a 78.7% of 5-year OS were identified in this cohort of patients	BC-Mps were insensitive to - NAC - routine chemotherapy - radiation therapy. BC-Mp is an aggressive type of breast cancer with poorer prognosis than IDC-NST and TN-IDC.
[32]	2,451 (2010–2014) Retrospective cohort study (data from national registry)	Luminal (19.1%) HER2+ (4.8%) TNBC (70.3%) Lack of data about exact IHC	No data about histology	T0 (0.2%), T1 (28.4%), T2 (45.2%), T3 (11.6%), T4 (5.4%), Tx (6%), N0 (78.5%), N1 (10.8%), N2 (2.4%), N3 (0.8%), Nx (5%) Lack of data about AJCC stage	CTH yes *n* = 1,816 (NAC *n* = 476) CTH no *n* = 611 For HR+ patients: ET yes *n* = 326; no *n* = 188	No data about CTH regimens.	Radiation and CTH were associated with improved OS
[15]	38 (2008–2013) Retrospective close cohort study	No precise data about IHC/BC in radical treatment	No precise data about histology in radical treatment	I (7.1%) II (57.15) III (23.8%) No precise data about T and N in radical treatment	CTH yes *n* = 29 (NAC *n* = 10); no *n* = 9 ET yes *n* = 17; no *n* = 21 NAC: A-based *n* = 2,Tx-based *n* = 1, A- + Tx-based *n* = 7 Adj CTH A-based *n* = 4, Tx-based 1, A-+Tx-based *n* = 11, TC *n* = 3	After NAC: CR 50%, PR 20%, SD 20%, PD 10% mOS = 38 mo	5-year OS was 76.3%. Better OS compared to series described earlier when - high grade, - large tumors, and half of them exhibiting nodal and hormonal involvement.
[23]	124 (2011–2020) Retrospective cohort study	Lack of precise data about IHC/BC in radical treatment	Lack of precise data about histology in radical treatment	I (3.7%) II (49%) III (34.8%) unknown (4.4%), No precise data about T and N in case of radical treatment	CTH yes *n* = 121 (NAC *n* = 41); no *n* = 3 ET yes *n* = 30 luminal cases TZB *n* = 14 Adj CTH regimens: A- and Tx-based *n* = 80, only A *n* = 20, Tx *n* = 14, X *n* = 5 NAC regimens: A- + Tx-based 2/3 patients, platinum-based *n* = 4	After NAC: pCR *n* = 3/41 (7.3%) mFU = 40 mo (range 2.6–130.8). The 5‐year DFS and OS were 56.4% and 57.6%, respectively.	BC-Mp is extremely resistant to typical CTH protocols. The worst OS was shown in: - spindle cell carcinoma, - pT3/4, - Ki67 > 45%, - TNBC, pN+.

A: anthracycline; AC: anthracycline, cyclophosphamide; A-T anthracyclines followed by taxane; AJCC: American Joint Committee on Cancer; BC-Mp: metaplastic breast cancer; BCSD: breast cancer-specific death; BCT: breast conservation therapy; CI: confidence interval; CMF: cyclophosphamide, methotrexate, 5-fluorouracil; CR: complete response; CTH: chemotherapy; CTX: cyclophosphamide; DM: distant metastases; DXL: docetaxel; DFS: disease-free survival; Epi: epirubicin; ER: estrogen receptor; ET: endocrine therapy; FAC: 5-fluorouracil, adriamycin, cyclophosphamide; FEC: 5-flurouracil, epirubicin, cyclophosphamide; mFU: median follow-up; G: gemcitabine; HER2; human epidermal growth factor receptor 2; HR: hazard ratio; IDC: infiltrating ductal carcinoma; IDC-NST: invasive carcinoma of no special type; IHC: immunohistochemistry; LRR: locoregional recurrence; LVI: lymphovascular invasion; M; metastases; MAID: mesnex, adriamycin, ifosfamide and dacarbazine; mOS/OS: median/overall survival; mo; months; N: nodules; N’: number of the study years; n: number of cases; NAC: neoadjuvant chemotherapy; NSABP: National Surgical Adjuvant Breast and Bowel Project; P; platinum; pCR: complete pathological response; PD; progressive disease; PSM: propensity score matching; PR: partial response; PrR: progesterone receptor; PXL: paclitaxel; RFS; recurrence free survival; RTH: radiotherapy; SD: stable disease; T: tumor; Tx: taxane; T+A combined taxane and anthracycline regimens; T/A; taxane and anthracycline regimen taxane or anthracycline regimen; TNBC: triple-negative breast cancer; TN-IDC: triple-negative invasive ductal carcinoma; TZB: trastuzumab; X: capecitabine.

Poor treatment outcomes for metastatic BC-Mp patients have previously been reported by our group [28]. The median DFS of 58.6 months and OS of 69.4 months in this study also suggest poor outcomes for BC-Mp patients treated with a radical approach (Tables 5 and 6). The majority of other studies confirm this poor prognosis, emphasizing the need for more effective therapies (see Table 9). The paper by Papatheodoridi et al. shows almost identical data for median DFS and OS: 56.8 and 66 months, respectively [24]. In the study by Song et al., the 5-year DFS and OS rates were 46% and 55%, respectively, which are similar to our findings (46% and 50%, respectively) [5]. However, in cohorts with a greater proportion of luminal patients with BC-Mp, the outcomes were more favorable [15, 21]. Additionally, in some studies, survival analysis indicated no noteworthy differences in DFS or OS between BC-Mp patients and IDC patients [12]. Approximately one-fifth of our patients developed distant metastases, typically within 1.5 years, consistent with findings from other studies [29]. One in ten patients presented with local recurrence, but interestingly, surgery did not influence OS. This underscores the necessity for vigilant patient monitoring during the initial 2-year follow-up period, particularly regarding the possibility of lung metastases and local relapse.

While the characterization of BC-Mp appears to be relatively consistent across different studies, the prognostic significance of individual characteristics, as well as the data on treatment efficacy, are divergent. In our multivariable analysis, only tumor size and the type of institution performing histopathological examination influenced DFS, while tumor size influenced OS.

The prognostic or predictive effect of BC-Mp histology was not detected in our cohort or in a few other studies [11, 17, 18, 27], but it was suggested by other authors [7]. We did not find a correlation between higher Ki67 levels, as indicated by Ismail et al. and Song et al. [5, 23], and patient outcomes. Numerous studies, including ours, confirmed an association between tumor size and DFS and/or OS [5], but in other patient cohort, such a correlation was not found [7]. Our findings did not demonstrate evidence that lymph node status influences patient prognosis, contrary to the suggestions of Song et al., Han et al., Zhang et al., and Erjan al. [5, 7, 21, 27]. The prognostic effect of BC-Mp histology was not detected in our cohort or in a few other studies [7, 21, 22, 30], but it was suggested by other authors [25]. Erjan et al. suggested that LV status can influence patient prognosis [21]. This was observed only in our univariable analysis.

The impact of the pathology facility where the initial diagnosis of BC-Mp is conducted (whether in reference/academic centra vs. nonreference/nonacademic centra) on treatment outcomes is an interesting finding. Youssef et al. observed such a correlation concerning treatment location [31], but we have not managed to find other papers that examined diagnosis location. It is possible that the factor responsible for this is a delay in treatment initiation when the diagnosis needs to be confirmed by a reference cancer centra.

There is a significant disparity in studies outcomes regarding the chemotherapy benefit for BC-Mp patients, with some papers confirming an association between systemic treatment and increased survival [32, 33] and other research not supporting this hypothesis [7, 8]. In our data, chemotherapy benefit was detected only in the univariable proportional hazards Cox model. Rakha et al. reported that chemotherapy correlated with extended survival although this association was observed predominantly in patients with early-stage disease [33]. It is also debated whether chemotherapy should be administered in the neoadjuvant setting or whether upfront surgery should be the preferred approach whenever feasible [11, 26]. Additionally, there is uncertainty about which regimens should be prioritized in this patient population. Yam et al. reported a 23% pCR rate in a population of 211 BC-Mp patients receiving NAC [16]. Another study based on the National Cancer Database performed by Haque et al. included more than 900 patients with BC-Mp who had a history of NAC and a pCR rate of 9.8% and suggested that early-stage patients have a greater probability of responding to treatment [34], which was not confirmed in our study. In the investigation conducted by Han et al., 29 patients, constituting 30% of the study cohort, underwent NAC, resulting in a pCR observed in five individuals, representing 17% of the treated population [27], similar to our outcomes where in *N* = 10 patients (20%) out of 49 with available data we have reported pCR (see Table 3). In a subset of 41 females from a cohort of 135 compiled by Zhang et al., anthracycline/taxane combinations were utilized in NAC settings, resulting in only three patients (7%) achieving pCR [7]. Wong et al. reported only one patient who achieved a pCR out of 44 patients who received NAC [6]. In a smaller population of 18 patients, Al-Hilli et al. reported a pCR rate of 11% [14]. Our current dataset represents one of the largest cohorts of patients undergoing NAC, with findings from a univariable Cox model demonstrating that taxane use (but not taxane and anthracycline combinations) improves OS. In multivariable Cox regression for the whole studied population (despite if systemic treatment was applied in neoadjuvant or adjuvant setting), the usage of taxane improved DFS, but not OS. Aydiner et al. reported that patients who underwent taxane-based chemotherapy regimens experienced improved OS [35].

As there are no separate guidelines regarding the treatment of BC-Mp, it is managed based on the stage and receptor status. The majority of these tumors are triple-negative breast cancer (TNBC) [5, 6, 9, 10, 12, 20–23]; therefore, a significant portion of patients qualify for neoadjuvant chemoimmunotherapy involving pembrolizumab [36]. According to the KEYNOTE-522 trial, while this study was not specifically focused on BC-Mp, the neoadjuvant treatment regimen included both taxane-based and anthracycline-based regimens administered concurrently with pembrolizumab [36].

Based on our database, it is difficult to draw conclusions regarding whether omitting radiotherapy or performing particular type of surgery affects the prognosis of patients, as surgery was employed in nearly all patients and radiotherapy was only omitted when it was not indicated. There are no specific treatment guidelines, including surgical or radiotherapeutic guidelines, for the management of BC-Mp other than those for IDC. Due to the suspected chemoresistance of BC-Mp, surgery is the primary therapeutic approach, emerging in some studies as an independent prognostic factor for patients with BC-Mp [10, 32]. There are limited data on the relationship between the extent of resection and patient survival. In some studies, breast-conserving therapy has been shown to be associated with a more favorable prognosis than mastectomy, as indicated by Kaplan–Meier OS curve for patients with BC-Mp [10, 37]. This may be caused by the effects of receiving radiotherapy following breast-conserving surgery and by the earlier disease stage of the primary tumor. Other studies have shown no difference in local recurrence, DFS or OS between patients who underwent BCS or mastectomy [10]. Lymph node sampling is advised regardless of the chosen surgical approach, and it is similar to IDC guidelines. In our group, the type of surgery was not associated with any difference in survival. It is crucial to emphasize that the extent of surgery may be difficult to assess, as our prior studies indicate that both ultrasound and mammography consistently underestimate the size of the primary tumor in BC-Mp patients [26].

The optimal radiotherapy schedule is challenging to determine due to the rarity of BC-Mp. Locoregional recurrence in BC-Mp patients after mastectomy may occur (in approximately 10% of our patients and in up to 28%–46% of patients in other studies); therefore, postoperative radiotherapy seems advisable in this group [5, 38, 39]. However, despite frequent relapses, the literature indicates that only 39% to 72% of patients with BC-Mp tumors receive postoperative radiotherapy, possibly due to the ambiguous results of published data [8, 32, 40–42]. Haque et al. demonstrated longer OS in a group of BC-Mp patients without distant metastases who received postoperative radiotherapy after BCT than in those who did not receive radiotherapy, regardless of the cancer stage and patient age. However, in the mastectomy group, the benefit of postoperative radiotherapy in terms of OS was observed only in patients with stage pT3–4/N+ disease [41, 42]. Tseng et al. showed that postoperative radiotherapy improved OS in BC-Mp patients after both breast-conserving surgery and mastectomy but had no effect on BC-specific survival [40]. Other researchers, however, observed that postoperative radiotherapy after mastectomy in patients with BC-Mp did not affect OS [33, 43]. The controversial status of postoperative radiotherapy in BC-Mp patients necessitates additional research to establish definitive guidelines due to conflicting outcomes in existing studies.

There is a pressing need for dedicated clinical trials focusing on BC-Mp. Currently, only a limited number of studies involve patients with BC-Mp, and furthermore, they predominantly target the metastatic population [44, 45]. An ongoing phase II trial (NCT05660083) is investigating the combination of an inducible nitric oxide synthase (iNOS) inhibitor and nab-paclitaxel along with alpelisib in patients with HER-2-negative, metastatic, or locally advanced metaplastic BC. Additionally, BC-Mp was investigated in a study (NCT02834013) involving nivolumab and ipilimumab in patients with various rare tumors. The phase 2 SABINA trial (NCT05810870) investigated MEN1611 (a phosphatidylinositol-4,5-bisphosphate 3-kinase catalytic subunit alpha [PIK3CA] inhibitor) alone and in combination with eribulin in HR+/HER2-negative metastatic BC-Mp patients with alterations in PIK3CA or phosphatase and tensin homolog (PTEN), assessing both its safety and efficacy. Our study revealed a remarkably high incidence of patients with prior malignancies in their medical histories (excluding those with concurrent active malignancies). It remains speculative whether the substantial number of tumor genetic alterations observed in BC-Mp [16, 18] may be linked to concurrent germline alterations.

### Study limitations

Important limitation of this study is its retrospective design. The low incidence of this neoplasm poses a challenge for prospective observation. Furthermore, the study acknowledges another constraint related to the size of the population. Nevertheless, it is noteworthy that our cohort is one of the largest published multicenter cohorts concerning BC-Mp patients containing detailed characteristic of studied population, treatment outcomes, and evaluating systemic treatment regimens applied. It is also one of the largest assessing patients, who received NAC. As BC-Mp was not formally acknowledged as a distinct histopathologic subtype until 2000, the classification was updated through the years and general trends towards application of NAC in lower BC stages are observed there is still limited information available on patient demographics, presentation, tumor characteristics, treatment patterns, and prognosis. Prudence is advised when interpreting data on the role of taxanes in the neoadjuvant setting, as the sample size of our groups was too small to create any recommendations. The data are derived from referral centra, and it is likely that worse outcomes are observed in regional centra given factors such as poorer diagnostics. Therefore, our results may not be fully representative. All our patients underwent surgery and received planned radiotherapy if required (in accordance with the guidelines for IDC). Due to the lack of a cohort of patients who did not receive radiotherapy (although they should have) or were not operated upon (although they qualified for the procedure), we cannot draw conclusions about the effectiveness of radiotherapy.

## Conclusions

We present real-world, multicenter data regarding the details of treatment received with an emphasis on systemic treatment for nonmetastatic BC-Mp patient populations. Notably, larger primary tumor size was significantly associated with poorer DFS and OS. Factors such as patient age, Ki67 status, molecular subtype, lymph node involvement, type of surgery, or receiving chemotherapy did not significantly impact DFS or OS. The efficacy of taxanes should be further explored, especially in the neoadjuvant setting. Our findings underscore the imperative for dedicated clinical trials in BC-Mp and tailored therapeutic strategies in this patient population [45].

## Supplementary Material

Treatment outcomes and prognostic factors in nonmetastatic metaplastic breast cancer patients: a multicenter retrospective cohort study

## Data Availability

The data are available upon reasonable request to the corresponding author.
